# Partially Resorbable Mesh Inclusion between Dissected Layers during Surgical Repair of Aortic Dissection

**DOI:** 10.1055/s-0041-1729919

**Published:** 2021-12-28

**Authors:** Corrado Cavozza, Tommaso Regesta, Antonio Campanella, Glauco Camporini, Andrea Audo

**Affiliations:** 1 Department of Cardiothoracic and Vascular Surgery, Cardiac Surgery, Santissimi Antonio e Biagio Hospital, Alessandria, Italy

**Keywords:** aortic dissection, partially reabsorbable mesh, aortic wall

## Abstract

Surgical management of aortic dissection is technically challenging for different reasons. Reapproximation of dissected layers because of fragility of the dissected aortic wall layers is of major concern. Many techniques have been described to restore the integrity of aortic wall. Inclusion of a partially resorbable mesh fixed with glue, between the dissected layers, may be a simple and effective method for providing a secure and viable end-to-end anastomosis between aortic stump and a Dacron graft.

## Introduction

Restoration of aortic wall integrity through reapproximation of the dissected layers represents a challenging surgical treatment. Problems are caused mainly by very friable aortic tissue damaged by dissection and by difficulties in obtaining sufficient mechanical strength during surgical reconstruction. 1

Many techniques have been reported to reinforce suturing between the aortic wall and a Dacron graft, while preventing anastomotic bleeding and avoiding creation of a new intimal tear. 2 3 4 5 6 7

Inclusion of a partially reabsorbable Polypropylene mesh with glue (BioGlue, CryoLife Inc., Kennesaw, GA), between the dissected layers, may represent a simple and effective method for providing a secure and viable anastomosis.

In addition to inserting a mesh ring trimmed to match the proximal aortic stump, we also reinforced the vessel by wrapping with Teflon felt strips or by adventitial inversion. A Dacron tube graft of appropriate size is cut obliquely and sutured to the transected aorta.

The suture should pass through all layers, with particular care being taken to include the intima. It is important to suture the graft to the aorta using two separate bites from inside to inside, crossing the stitch to discourage tearing of the aorta.

## Technique and Results



**Video 1** A strip of mesh is trimmed to affected area and then put between the dissected layers and fixed with surgical adhesive. Bulldog clamps are applied to aortic stump for some minutes, and glue through large pores converts the layers into a more resistant texture, incorporating the surgical adhesive.


All patients gave permission to publish their information and images.

During the past year, eight patients with Type A aortic dissection underwent surgical treatment with the aid of this technique. Surgically relevant variables and aortic morphology were evaluated by computed tomography. Supracoronary graft replacement was performed in all patients.

The aorta was reinforced with Teflon felt placed external to the adventitia and internal to the intima, with inclusion of polypropylene mesh for both the proximal and distal aortic stumps. The use of hernia mesh to surgically repair or reconstruct anatomical defects has been widely adopted in general surgery.

The mesh in this study was Ultrapro (Ultrapro, Ethicon, Somerville, NJ), a large pore composite mesh (polypropylene and poliglecaprone, Monocril; weight = 28 g/m ^2^ , pore size 3 mm).

**Fig. 1:**
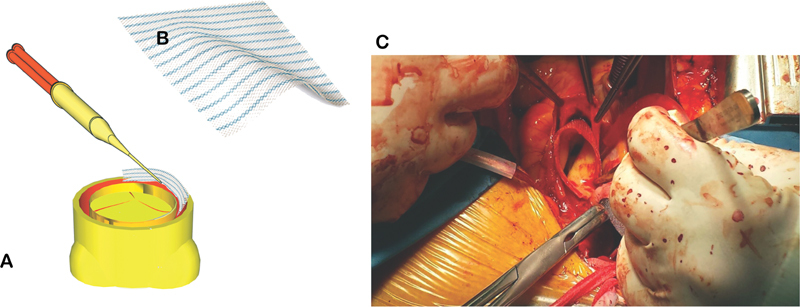
( **A** )Hernia mesh, which has already been trimmed to match the affected area, is placed between the dissected layers and fixed with glue. Mesh in proximal aortic stump with application of glue. ( **B** ) Hernia mesh. ( **C** ) Intraoperative view.

Hernia mesh, which had already been trimmed to match the affected area, is placed between the dissected layers and fixed with glue ( Fig. 1 ; Video 1 [available in the online version]). Application of the adhesive through the large pores not only produces approximation of the dissected wall layers but also converts them to a more resistant texture.

Then a Dacron tubular graft is sutured end-to-end to the aortic proximal and distal stumps using continuous suture. Every surgical suture compresses the enclosed tissue with tension adjusted depending on the feel of the tissue and suture. Over the decades, despite considerable technical improvements, failure of surgical sutures, and tissue leads to relevant complications such as new intimal tear and anastomotic bleeding.

Until now, surgeons have had no other criteria besides their purely subjective “feeling” of what the tissue needs in terms of suture tension and avoiding further local tissue damage. Due to the limited visual control in the process of suturing, the surgeon has to rely on their firm belief that their suture technique and suture tension are “appropriate” for the tissue.

Furthermore, the range of the tension considered as “appropriate” shows wide overlap with too high or too loose tension. Because of missing precise data to define the optimum suture tension, surgical suture repair is based mainly on an individual feeling for suture tension.

**Fig. 2:**
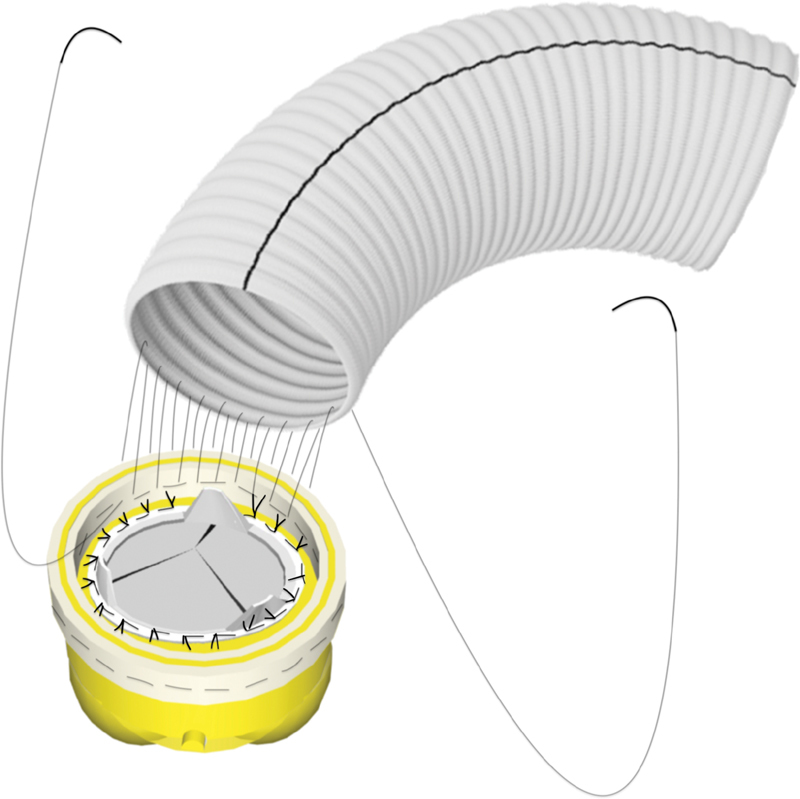
Technique for end-to-end Dacron aortic anastomosis. After the aortic wall has been reinforced, a tube graft is held opposite to the transected aorta. The anastomosis is performed with a continuous suture, the needle of which goes through the aorta (in–out), through the graft (in–out), and again through the aorta (in–out).

We start the running suture with the graft. The anastomosis is made with a continuous suture, the needle of which goes through the aorta (in–out), through the graft (in–out), and again through the aorta (in–out; Fig. 2 ). This surgical approach, making this technique particularly safe, reduces significantly the need for hemostatic stitches after completion of the anastomosis.

Aortic regurgitation was treated in this series by resuspension of the aortic commissures.

The mean age of treated patients was 72 ± 6 years. Cardiopulmonary bypass time was 190 ± 45 minutes. Lower body circulatory arrest time was 28 ± 10 minutes. There was one hospital death. The mean postoperative blood loss was 650 ± 220 mL, and one reexploration was required.

Postoperative computed tomography at 6 months showed closure of the false lumen in the aortic root, aortic arch, and proximal descending thoracic aorta in all surviving patients. Postoperative echocardiography demonstrated minimal or no aortic regurgitation in all patients.

## Discussion

Surgical treatment of aortic dissection remains challenging, although several strategies have been adopted to reinforce the fragile aortic wall layers and to enhance hemostasis. 1 Classic ascending aortic replacement techniques include reinforcement of the proximal aortic stump with Teflon felt or pericardial patch. Teflon felt is adopted when performing the sandwich technique or it can also be used as neomedia between the dissected layers. 2 Furthermore, various types of surgical glues are available to facilitate repair of the aorta and to reduce bleeding from the anastomosis. Regardless of the surgical technique adopted to reinforce the aortic wall and to perform the anastomoses, the aim is always to obliterate the false lumen. Prosthetic materials for the repair of abdominal wall defects have been studied extensively to improve outcome. A new approach can be the use of a synthetic mesh, usually adopted in hernia repair.

UltraPro (Ethicon, Somerville, NJ), a partially absorbable mesh overcomes the disadvantages of both heavyweight and ultralight mesh, consisting of an entanglement of absorbable and nonabsorbable fibers; it offers feasibility, strength in the early postoperative period, and foreign body materials left behind, allowing an optimal biocompatibility and incorporation into the host tissue. 8

Partially reabsorbable mesh into the false lumen acts as neomedia and enables a fine layer of glue spread within the false lumen. After its application, the dissected membrane should be aligned and held firmly to the outer aortic wall to ensure a uniform bonding. The low viscosity of BioGlue allows leakage through the mesh holes and induces serum albumin, extracellular matrix proteins, and cell surfaces to bind to each other creating a strong scaffold that reinforces the anastomosis with the Dacron graft. The running suture is started with the graft. The anastomosis is made with a continuous suture, the needle of which goes through the graft (in–out), through the aorta (in–out), and again through the graft (in–out).

No pseudoaneurysms or intimal tears or other anastomotic complications were observed at late follow-up with computed tomography scan. These results are obviously limited by the small sample size.
